# Transiently chaotic simulated annealing based on intrinsic nonlinearity of memristors for efficient solution of optimization problems

**DOI:** 10.1126/sciadv.aba9901

**Published:** 2020-08-14

**Authors:** Ke Yang, Qingxi Duan, Yanghao Wang, Teng Zhang, Yuchao Yang, Ru Huang

**Affiliations:** 1 Key Laboratory of Microelectronic Devices and Circuits (MOE), Department of Micro/nanoelectronics, Peking University, Beijing 100871, China.; 2 Center for Brain Inspired Chips, Institute for Artificial Intelligence, Peking University, Beijing 100871, China.; 3 Frontiers Science Center for Nano-optoelectronics, Peking University, Beijing 100871, China.

## Abstract

Optimization problems are ubiquitous in scientific research, engineering, and daily lives. However, solving a complex optimization problem often requires excessive computing resource and time and faces challenges in easily getting trapped into local optima. Here, we propose a memristive optimizer hardware based on a Hopfield network, which introduces transient chaos to simulated annealing in aid of jumping out of the local optima while ensuring convergence. A single memristor crossbar is used to store the weight parameters of a fully connected Hopfield network and adjust the network dynamics in situ. Furthermore, we harness the intrinsic nonlinearity of memristors within the crossbar to implement an efficient and simplified annealing process for the optimization. Solutions of continuous function optimizations on sphere function and Matyas function as well as combinatorial optimization on Max-cut problem are experimentally demonstrated, indicating great potential of the transiently chaotic memristive network in solving optimization problems in general.

## Teaser

This work shows a memristive optimizer based on transient chaos, which is able to solve optimization problems efficiently.

## INTRODUCTION

People are inevitably facing various optimization problems at all times to improve efficiency, use resources rationally, and find the best solution under certain constraints. For example, combinatorial optimization problems, including the traveling salesman problem (TSP), knapsack problem, and graph coloring problem (*1*), have very simple descriptions but can represent a lot of practical engineering problems. A large variety of complex optimization problems are raised from both traditional and emerging application domains, including scheduling and planning problems, logistics and transportation, smart factories, and engineering design. Meanwhile, heuristic algorithms inspired by human intelligence, animal society, and physical phenomena, such as artificial neural network, genetic algorithm, ant colony algorithm, and simulated annealing (*2*), have been developed to tackle these problems.

Among these approaches, the Hopfield network (*3*) can solve optimization problems by minimizing its energy function during network evolution and has been considered suitable for efficient hardware implementation because of its simple computing elements and parallel computing process. The Hopfield network falls into the category of recurrent neural networks (RNNs), which has different dataflow from feedforward neural networks (FNNs). In FNNs, the data processed by one layer will be passed to the next cascaded layer persistently until the final result is obtained. In contrast, the output from one layer is connected to the input of exactly the same layer at the next time step in RNNs. During network evolution, the energy of the Hopfield network spontaneously decreases, and the network gradually converges toward the stored attractors that can be decided by the weight matrix, therefore allowing the network to implement associative memory (*4*) and solve optimization problems (*3*). Combination of simulated annealing with Hopfield networks could further help the network jump out of local minima and find a better or even optimal solution (*5*).

Memristor, as the abbreviation for “memory resistor,” is able to retain the memory of external electrical stimulation history in its physical state (*6*). In general, the memristor device has a simple two-terminal structure and thus can be easily integrated into a high-density crossbar structure. This compact memristor crossbar can be used to implement vector-matrix multiplication (VMM) efficiently based on Ohm’s law and Kirchhoff’s current law (*7*), which is promising in building non–von Neumann computing architecture that avoids frequent data transport. This has led to the flourish of memristor-based accelerators for a large variety of algorithms that involve heavy VMM operations (*8*, *9*). Among them, memristor-based hardware for FNNs, including perceptron (*10*) and convolutional neural networks (*11*), has been studied extensively, and software-equivalent accuracy with high efficiency has been reported (*12*). Besides, other neural network hardware based on memristive devices have also been demonstrated, including long short-term memory (*13*), reinforcement learning (*14*), and Hopfield networks for associative memory (*15*–*17*). In addition to these typical neural networks, other algorithms such as image processing (*18*), solution of partial differential equations (*19*), and solution of matrix equations (*20*) have been implemented in memristive hardware as well.

Despite the encouraging progress, only very limited studies have exploited the potential of memristive hardware in solving optimization problems. Among them, the first demonstration on memristor-based optimizer was an analog-to-digital conversion using discrete devices, where memristors were used to store the weights in Hopfield networks (*21*, *22*). Furthermore, by combining the VMM acceleration enabled by a memristor array with the stochasticity of devices, stochastic simulated annealing (SSA) strategy can be introduced into the solution of optimization problems (*23*, *24*). It was reported that a spin glass problem can be mapped and solved on a memristor crossbar along with extra conductive bridge random access memory cells for decision of the spin-flip event (*23*). In another study, the intrinsic random telegraph noise in memristor array was used as a random signal source (*24*), which enables simulated annealing via modulating signal-to-noise ratio controlled by periphery circuits. It usually requires either additional devices or sophisticated periphery circuits besides the memristor crossbar itself to realize solution of optimization problems, which compromises the advantage offered by compact memristor array to a certain extent. Fortunately, the dynamics of the network can also be manipulated to realize simulated annealing. That is, a Hopfield network can undergo a transition from chaotic wandering to convergence when its self-feedback weights are adjusted, hence leading to a chaotic simulated annealing (CSA) strategy (*25*, *26*). This transition can be triggered by storing the self-feedback weights in an additional memory array (e.g., NOR flash or memristor) and scaling down the input voltages during runtime (*25*, *26*), demonstrating a practical route toward memristor-based CSA systems.

In this study, we report a compact CSA optimizer based on a single Ta/TaO*_x_*/Pt memristor crossbar array after an equivalent mathematical transformation of the algorithm, where efficient nonlinear annealing with high probability of global optimum and fast convergence can be achieved via programming diagonal devices in the array with simple pulse schemes. The weight matrix of a Hopfield network is mapped onto the crossbar, and the conductance values of diagonal memristors are consistently decreased to trigger the transition from chaotic searching to convergence. Experimental demonstrations show that these transiently chaotic networks can solve continuous function optimizations, taking sphere function and Matyas function as two examples, as well as combinatorial optimization such as Max-cut problem. The intrinsic nonlinearity of the diagonal memristors when programmed by identical pulses offers key dynamics to the transiently chaotic network, which is proved to be an efficient and simplified annealing strategy compared with linear and exponential annealing processes. The transiently CSA hardware demonstrated in this study holds great potential in efficient solution of optimization problems in general.

## RESULTS AND DISCUSSION

### Memristor-based transiently chaotic Hopfield network

**Fig. 1:**
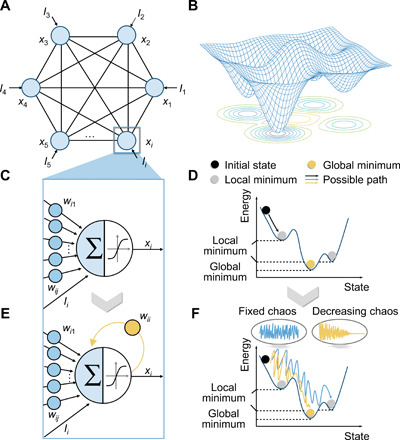
Illustration of a Hopfield network with transiently chaotic dynamics. (**A**) Schematic of a fully connected Hopfield network. (**B**) Illustration of the network energy surface. A Hopfield network generally contains energy minima as attractors. (**C**) Standard neuron of a Hopfield network, which sums internal inputs from other neurons and external bias and then generates the output through an activation function. (**D**) Possible state evolution of a standard Hopfield network. When the initial state is near a local minimum, the network can be easily trapped (black line). (**E**) Single neuron of a Hopfield network with self-feedback. The output of neuron *i* is recurrently connected to itself, scaled by the self-feedback weight *w_ii_*. (**F**) Schematic of state evolution of a Hopfield network with transient chaos. When chaos is introduced into the network with constant self-feedback weight, the network may get out of local minima but is hard to converge (blue line). While using the transient chaos, the network may converge toward the global minimum (yellow line).

A Hopfield network (*27*) is a single-layer artificial neural network, whose neurons are fully connected with each other (Fig. 1A), and has a bumpy energy surface with many local extremes in general (Fig. 1B). It is worth noting that a classic Hopfield network always restricts its weight matrix to be symmetric without nonzero elements in the diagonal positions, which means that the neurons have no self-feedbacks (as shown in Fig. 1C) to keep the network stable and ensure its convergence. This stability decides that the network has a chance to fall into and stay at local minima near the initial states (as shown in Fig. 1D), which is the key mechanism for realizing associative memory. However, this is undesirable for the solution of optimization problems.

To address this issue, we hereby introduce transient chaos into the network by adding proper self-feedbacks to the neurons (Fig. 1E), which is expected to help the network jump out of the local minima (Fig. 1F) (*5*). Nevertheless, if the network is shaken persistently by the chaos, the network may have difficulties in reaching convergence. We have therefore introduced a transient chaos into the memristive network by gradually reducing the self-feedback weights during the iterations in the present study, which can help the network find and get stabilized at the energy minimum. Specifically, the above CSA process based on a Hopfield network can be described by the following equations (*5*)

$$x_{i}(t)=\frac{1}{1+e^{-y_{i}/\varepsilon }}$$ (1)

$$y_{i}(t+1)=ky_{i}(t)+\alpha (\sum_{j=1}^{n}w_{\mathrm{ij}}x_{j}(t)+I_{i})-z_{i}(t)(x_{i}(t)-I_{0})$$ (2)

$$z_{i}(t+1)=(1-\beta )z_{i}(t)$$ (3)

where the three time-dependent variables *x_i_*, *y_i_*, and *z_i_* are defined as the output, the internal membrane potential, and the self-feedback weight of neuron *i*, respectively. The neuronal output *x_i_* can be calculated by *y_i_* through the sigmoid function with steepness parameter ε. Equation 2 describes the iteration of the membrane potential, which contains three items. The first part is the leaky item, which means that the neuronal history is memorized with a damping factor *k*. The second item represents the input to neuron *i* at time *t*, including the collective influence by the other neurons and the external stimuli *I_i_*, which is scaled by a positive parameter α. The last item is the newly introduced self-feedback aiming to endow the network with transiently chaotic dynamics, where *I*_0_ is a positive parameter. To make the chaotic state transient, the self-feedback weight needs to be weakened over time, and Eq. 3 defines the annealing process, which can be an arbitrary function, such as linear or exponential annealing. As an example, Eq. 3 shows an exponentially decayed self-feedback with a damping parameter β.

**Fig. 2:**
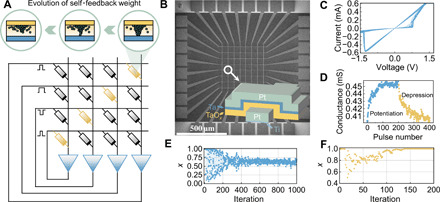
Mapping a Hopfield network with transient chaos onto a single memristor array. (**A**) Illustration of a Hopfield network with transient chaos mapped on the memristor crossbar. The devices in diagonal positions represent self-feedback weights, and the other devices map the constant parameters in optimization tasks, which stay unchanged once the task is determined. Neuronal inputs are translated into voltage pulses with different widths and applied onto the rows of the array, while the output currents are collected in each column. The function of neuron is implemented in software. (**B**) Scanning electron microscopy image of the Ta/TaO*_x_*/Pt memristor array. The inset illustrates the stacking structure of the device. (**C**) *I-V* characteristics of the device repeated for 10 cycles. (**D**) Long-term potentiation/depression characteristics of the device in response to 200 identical pulses for potentiation (1.4 V, 100 μs) and depression (−1.25 V, 2 μs), respectively. (**E**) Dynamic behavior of a single chaotic neuron without bias. The neuronal output converges to around 0.65 after chaotic searching. (**F**) Dynamic behavior of a single chaotic neuron with bias (α*I*_i_ = 0.005). The neuronal output is attracted to 1 after chaotic searching.

Implementation of this algorithm in complementary metal-oxide semiconductor (CMOS) circuits can be expensive in time, area, and power consumption. As this network is fully connected, the number of matrix parameters will grow in a quadratic manner with the network size and hence result in excessive VMM operations. Moreover, implementation of such VMM operations in traditional von Neumann architecture requires frequent data movement from off-chip memory (*28*, *29*), and complex operations involved in Eq. 3, such as the exponential function, are also difficult to be realized in a CMOS circuit (*30*). In the present transiently CSA hardware based on memristors, the multiplication and accumulation operations as well as the expensive exponential function generation are both accomplished in situ using a compact memristor crossbar, as shown in Fig. 2A. The memristor array used here is based on a Ta/TaO*_x_*/Pt structure, with each device sandwiched at the cross-point between the top and bottom electrodes, as shown in Fig. 2B. Detailed analysis on the microstructure of the devices can be found in the transmission electron microscopy (TEM) images and energy-dispersive x-ray spectroscopy (EDS) characterization shown in figs. S1 and S2. Current-voltage (*I-V*) characteristics of the devices shown in Fig. 2C exhibit stable bipolar resistive switching with little cycle-to-cycle variation. Figure 2D further shows the long-term analog response of the Ta/TaO*_x_*/Pt devices during potentiation and depression processes (Fig. 2D), which is crucial for the realization of CSA afterward.

To map the abovementioned algorithm onto the memristor array, the weight matrix corresponding to specific optimization tasks is programmed into the conductance values of the memristor array element-wisely (black devices in Fig. 2A). The conductance of each device represents a synaptic weight through a linear transformation *G_ij_* = *aw_ij_* + *b*. A write-and-verify strategy is used to program the selected devices, where programming voltage pulses are applied on the top electrodes, with the bottom electrode grounded. In particular, the self-feedback weights of Hopfield neurons are mapped onto the diagonal devices within the array (yellow devices in Fig. 2A), which are responsible for achieving transient chaos. Note that there is a difference between the input applied to the self-feedback weight and that applied to the other devices on the same row according to the mathematical model described in Eq. 2. We have therefore adopted a mathematically equivalent transformation to the model, which allows the VMM and the annealing to be performed within the same crossbar (see Materials and Methods for details).

**Fig. 3:**
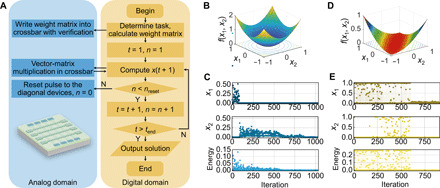
Experimental solution of continuous function optimizations with transiently chaotic networks. (**A**) Operation flowchart of the transiently chaotic network based on Ta/TaO*_x_*/Pt memristors. (**B**) Surface of the sphere function with its minimum at point (0, 0). (**C**) Experimental solution of the sphere function optimization with the transiently chaotic network based on Ta/TaO*_x_*/Pt memristors, showing the evolutions of neuronal outputs and corresponding Hopfield energy. (**D**) Surface of the Matyas function. (**E**) Experimental solution of the Matyas function optimization with the transiently chaotic network based on Ta/TaO*_x_*/Pt memristors, showing the evolutions of neuronal outputs and corresponding Hopfield energy.

After mapping the algorithm onto the hardware, the memristive optimizer can be used to solve optimization problems. The whole optimization procedure on the proposed memristive optimizer consists of two phases, which are named as the update phase and the program phase, respectively (Fig. 3A). In the update phase, the neuron outputs at time step *t* are converted into voltage pulses, whose pulse widths are proportional to *x_i_*(*t*), which are applied recurrently onto the *i*th row as inputs, while the columns are virtually grounded. Following this setup, VMM can be obtained physically in a single read cycle, because the charge integrated at column *i* is proportional to $\sum_{j=1}^{n}G_{\mathrm{ij}}x_{j}(t)$ according to the Ohm’s law and Kirchhoff’s current law. Note that the self-feedback has been included here. This intermediate result is then transferred into software to get the updated membrane potential as well as the neuronal output. In the subsequent program phase, depression pulses are applied to the top electrodes of diagonal memristors to reduce the self-feedback weights according to predefined Eq. 3. Note that, here, the self-feedback weight is not programmed every time but in every *n*_reset_ iteration to further speed up the operation of the algorithm in hardware.

If the size of the network is reduced to one, the network is essentially a single transiently chaotic Hopfield neuron with self-feedback, serving as the building block of the proposed network. Figure 2E shows the characteristics of a single transiently chaotic neuron, where the neuronal output displays evidently chaotic property in the early stage and gradually converges to around 0.65 as the self-feedback weight persistently decreases, showing the expected transition from chaos to convergence. It is worthwhile pointing out that solution of optimization problems requires such a Hopfield neuron to be capable of tracing energy minimum in different energy landscapes. As expected, when a positive external bias is applied to the neuron (hence, the energy function is −*Ix*), the neuronal output approaches one instead after the chaotic stage so that the energy function can be minimized, given the searching space of (0, 1) of Eq. 1, as illustrated in Fig. 2F. This therefore demonstrates the applicability of the present transiently chaotic neuron to the solution of optimization problems.

### Solution of continuous optimization problems using transiently chaotic memristive network

The above memristive optimizer with transiently chaotic dynamics can be used to solve typical optimization problems, taking continuous function minimization as an example, which is an important type of optimization problems with continuous variables. Optimization problems with discrete variables are categorized into combinatorial problems and will be discussed next. Here, we experimentally demonstrate the potential of transiently chaotic memristive networks in solving continuous function optimizations, taking a simple sphere function $f(x)=x_{1}^{2}+x_{2}^{2}$ as an example first, where the goal is to find the minimum of the function, namely, (0, 0) in the present case (Fig. 3B). The target function is mapped to the network weights through energy function so that the solution can be found when the energy converges to its minimum. Therefore, the energy function is assigned to be $x_{1}^{2}+x_{2}^{2}$, with *x*_1_ and *x*_2_ as the outputs of two neurons. These two neurons are connected with each other by a 2 × 2 weight matrix, which is implemented in a 2 × 2 sub-array of the memristor crossbar. The weights are extracted by taking the derivative of the energy function $\sum_{j=1}^{n}w_{\mathrm{ij}}x_{j}+I_{i}=-\partial E/\partial x_{i}$ and programmed subsequently into the memristor array based on linear transformation with a write-and-verify scheme. The programming stops once the device conductance has a relative difference of <10% compared with the target value. After the weights are successfully mapped, the abovementioned iteration including update and program phases starts, with the self-feedback weight programmed after every 10 iterations, i.e., *n*_reset_ = 10. The self-feedback is initialized to 0.08, a value selected to ensure sufficient chaotic searching and reasonably fast convergence (as discussed in detail in fig. S3), and the membrane potentials of both neurons are initialized to a median value of 0.5 in the present case. The above update and program iterations proceed until the network converges to the final optimized solution.

Figure 3C presents the experimental result of optimization using the transiently chaotic network, showing that the strong self-feedbacks in the initial stage result in chaotic wandering in the solution space. As the self-feedback persistently decreases when the diagonal memristors are programmed, the network starts to converge after hundreds of iterations, and the solution (0, 0) is successfully obtained eventually, hence demonstrating the capability of the proposed approach in solving continuous optimization problems. It could be noticed that no negative values have been wandered in the solution space (Fig. 3, B and C), which can be attributed to the output range of (0, 1) of the activation function used in Eq. 1 but is sufficient to solve the sphere function optimization problem because of the symmetry of the function with respect to *x*_1_ and *x*_2_ axes. However, if the solution is beyond the searching space offered by the sigmoid function, it will be necessary to use a different activation function with larger output range (e.g., tanh function with an output of −1 to 1), or instead, the variables of the target function can be shifted and/or scaled to match the searching space with the solution space.

The applicability of the present approach can be extended to other continuous optimization problems following the same protocol. To verify the general applicability of the present approach, we have performed additional experiment on the minimization of Matyas function, as illustrated in Fig. 3 (D and E). The Matyas function is defined as $f(x_{1},x_{2})=0.26(x_{1}^{2}+x_{2}^{2})-0.48x_{1}x_{2}$, which has a plate-shaped surface with small gradients along the bottom and global minimum at (0, 0) (Fig. 3D). One can see that the two neurons have converged to around (0, 0) after chaotic wandering, while the Hopfield energy approaches the minimum of the Matyas function, once again solving the problem correctly. Therefore, the successful minimizations of sphere function and Matyas function experimentally demonstrates that the transiently chaotic Hopfield network based on memristors can be used to solve continuous optimization problems in general.

### Solution of combinatorial optimization problems using transiently chaotic network

**Fig. 4:**
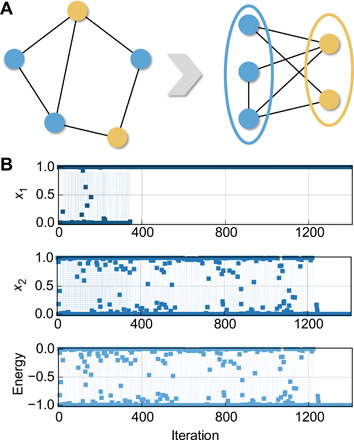
Experimental solution of combinatorial optimization problems with transiently chaotic networks. (**A**) Schematic of the Max-cut problem. The given vertices need to be separated into two different groups while maximizing the edge across. (**B**) Experimental solution of the Max-cut problem with the transiently chaotic network based on Ta/TaO*_x_*/Pt memristors, showing the evolutions of neuronal outputs and corresponding Hopfield energy.

Besides continuous function optimizations, the applicability of the present approach may be further extended to combinatorial optimization problems as well. Here, we take the Max-cut problem as an example to experimentally demonstrate the capability of our approach in solving combinatorial optimization problems, which is one of the most important non-deterministic polynominal (NP)–hard combinatorial problems widely used in network optimization, statistical physics, and very large-scale integration (VLSI) designs (*31*). As illustrated in Fig. 4A, in a Max-cut problem, the goal is to find a segmentation for this graph so that all vertices can be divided into two groups while maximizing the number of edges that are cut through. To map this problem onto the network, each neuronal output is assigned as the classification result of the corresponding vertex. Namely, a neuronal output of 1 indicates that this vertex is assigned to the first group, while a neuronal output of 0 means that the vertex belongs to the other group. Mathematically, the target is equivalent with maximizing the following score, where *a_ij_* equals 1 if there is an edge between vertices *i* and *j* (otherwise equals 0)

$$\text{score}=\sum_{i<j}a_{\mathrm{ij}}\frac{1-(2x_{i}-1)(2x_{j}-1)}{2}$$ (4)

Mapping into the Hopfield network, the task is equivalently transformed to minimize the energy function in the following expression

$$E=\sum_{i<j}a_{\mathrm{ij}}(2x_{i}x_{j}-x_{i}-x_{j})$$ (5)

Thus, the weight accordingly should be set to

$$w_{\mathrm{ij}}=a_{\mathrm{ij}}$$ (6)

We transposed a two-node Max-cut problem with one edge to the memristor hardware using 2 × 2 array for proof of concept, which has three possible solutions in this simple case. These two nodes may be assigned to the same group (group 1 or group 2) or different groups. Obviously, the latter scenario can earn one score and therefore is the optimal solution to the problem. The initial membrane potentials of both neurons are random values within the range of ( − 1,1), and the self-feedback weights are initialized to 0.077. Figure 4B shows the experimental result of optimization using the transiently chaotic network, where neurons 1 and 2 have converged to 1 and 0, respectively, after chaotic wandering (Fig. 4B). The different iteration cycles for the two neurons before convergence may be caused by device-to-device variations. Fortunately, the network still finds the optimal solution with such variations, showing the robustness of the computing system. The energy evolution shown in Fig. 4B characterizes the state of the network, implying that optimal solution has been found eventually. Despite a still small-scale problem, the memristor-based transiently chaotic network has once again solved the combinatorial optimization problem and may be extended to more complex cases in principle.

### Harnessing the intrinsic nonlinearity of memristors for efficient and simplified optimization

**Fig. 5:**
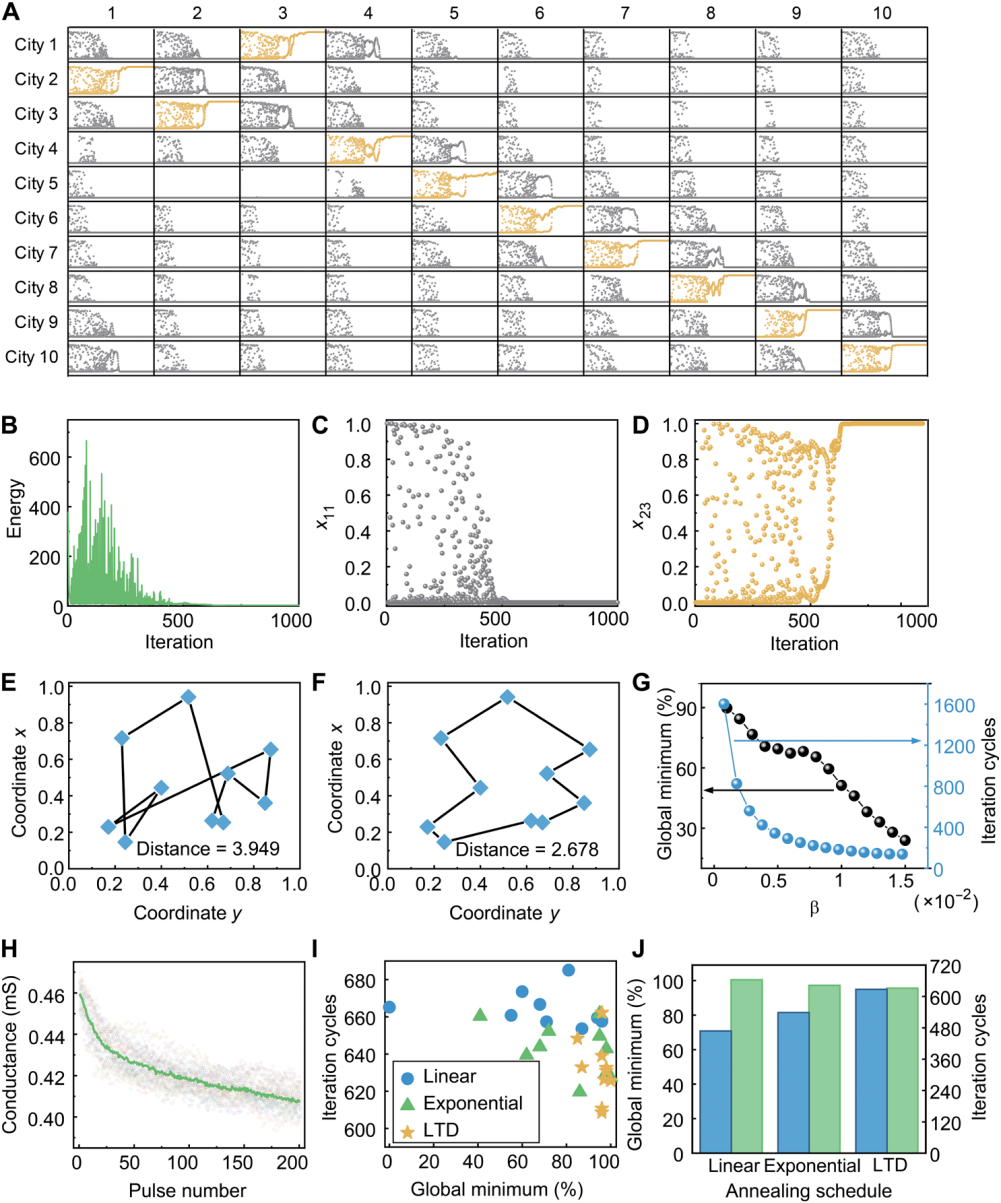
Efficient and simplified annealing based on intrinsic nonlinearity of memristors. (**A**) Simulation result for the evolution of the Hopfield network when solving the 10-city TSP problem. The numbers of row and column represent the city and visiting sequence, respectively. The activated neurons are depicted in yellow. (**B**) Energy evolution of the transiently chaotic network. (**C**) Evolution of output for an unactivated neuron *x*_11_. (**D**) Evolution of output for an activated neuron *x*_23_. (**E**) Randomly selected traveling route for the TSP problem in question. (**F**) Optimized traveling route after solution with transiently chaotic network. (**G**) Probability of getting global minimum and iteration cycles as a function of β when exponential annealing process is adopted. (**H**) LTD process of the Ta/TaO*_x_*/Pt device in response to identical programming pulses (−1.25 V, 2 μs) repeated for 20 cycles. The line indicates the averaged curve. (**I**) Comparison of linear, exponential, and LTD annealing processes. Each point corresponds to a randomly generated city graph, showing averaged iteration cycles and the probability of finding global optimum from 1000 simulations, i.e., 100 random initial conditions for 10 randomly generated city graphs. (**J**) Corresponding statistical analysis on the probability of finding global optimum (blue) and average iteration cycles (green).

The successful solutions of two continuous optimization problems, including sphere function (Fig. 3, B and C) and Matyas function (Fig. 3, D and E), as well as combinatorial optimization problem such as Max-cut (Fig. 4) have unambiguously shown the potential of the proposed network in efficient solution of optimization problems. We have performed further simulations on a typical combinatorial optimization problem in a larger scale, namely, 10-city TSP. Figure 5A shows a typical evolution of the Hopfield neurons in the network, with the energy evolution shown in Fig. 5B. Figure 5 (C and D) further illustrates detailed evolutions of an unactivated neuron and an activated one, respectively. Notably, a shortest distance of 2.6776 is obtained after chaotic searching (Fig. 5E), which is obviously better than a random solution (Fig. 5F). Figure 5G compares the probability of getting global minimum and iteration cycles when exponential annealing process is adopted. One can see that, although the convergence speed is improved when β is increased, the probability of finding global minimum decreases as well, forming a dilemma in selecting an appropriate β value.

The above transiently chaotic Hopfield network for solution of optimization problems shares a similar concept with simulated annealing (*32*), which is inspired by the physical annealing of materials and is widely used in optimization algorithms. While materials can usually find their energy minima in crystalline states when the annealing temperature drops slowly, they may end up with an amorphous state with high energy if rapid cooling is used. Similarly, the annealing process is also fundamentally important in the present case to ensure that the global minimum can be obtained with high probability and efficiency. In particular, an infinitely slow annealing process does not necessarily offer a global optimum in the transiently chaotic networks. Instead, the result is decided by the global bifurcation structure of the network, and hence, a proper annealing strategy will be essential for efficient solution of optimization problems. This annealing process is implemented by the detailed depression curve of memristive devices sitting in diagonal positions of the crossbar array (Fig. 2A), which decides how the computing system dynamically searches in the solution space and get stabilized afterward.

Typically, linear weight update is desirable in online training of artificial neural network accelerators based on memristors (*33*), but oxide-based memristors usually exhibit nonlinear potentiation/depression in response to identical voltage pulses as shown in Fig. 2D due to their intrinsic switching mechanism (*34*, *35*). In the present case, the purpose of the modulation on diagonal weights is not for online training but to tune the dynamics of the network so that it can trigger a transition from chaotic wandering to convergence, therefore offering the substrate for solution of optimization problems. Although such nonlinearity in weight modulation is widely considered a challenge for online training (*36*–*39*), it may become favorable here for the transiently chaotic dynamics, as will be shown below.

We have studied and compared three different annealing processes, i.e., linear, exponential, and intrinsic long-term depression (LTD) processes in memristors (Fig. 2D). These different decay curves can be achieved by designing the number of pulses (with fixed amplitude and width) applied at each step based on known depression characteristics of the devices in response to identical voltage pulses, as shown in detail in fig. S4. While LTD annealing can be naturally achieved simply by applying identical voltage pulses (Fig. 2D), both linear and exponential annealing processes require modulation on the number or width of voltage pulses. Figure 5H further shows the LTD process of Ta/TaO*_x_*/Pt devices in response to a train of identical programming pulses (−1.25 V in amplitude, 2 μs in width, 4 ms in interval) repeated for 20 cycles, where the averaged experimental data can be fitted by a double exponential decay function *a* × e^−*bx*^ + *c* × e^−*dx*^ (fig. S4B). We have conducted simulations on 10 randomly generated city graphs using linear, exponential, and LTD annealing processes, and each simulation for a specific TSP graph was performed for 100 different initial conditions, namely, 1000 rounds in total for each annealing strategy (figs. S5 and S6). As shown in Fig. 5I, the points corresponding to nonlinear annealing processes, including both exponential and LTD annealing, are distributed in the lower right corner, implying higher probability in obtaining global minimum and less iteration cycles compared with linear annealing. Figure 5J further illustrates averaged probability of finding global minimum and averaged iteration cycles for linear, exponential, and LTD annealing processes, respectively, where one can clearly see the overall best performance of LTD annealing. The implementation of LTD annealing can be facilely achieved by applying identical voltage pulses (fig. S4), therefore holding great advantage.

These different efficiencies obtained in linear, exponential, and LTD (i.e., double exponential) annealing processes may be understood by their different cooling speeds. The CSA system is primarily dependent on the global bifurcation structure of the transiently chaotic networks, and therefore, unlike SSA, an infinitely slow annealing does not necessarily lead to an optimum result (*40*). The reason is that the optimum solution does not always survive after the coexisting chaotic attractors eventually merge into a single global attractor (*40*). As a result, the CSA generally requires adaptive annealing speed, and the LTD annealing (i.e., double exponential) turns out to be a proper dynamic process.

Note that CSA based on transiently chaotic dynamics is totally deterministic in theory, which is implemented by tuning diagonal weights of the crossbar and hence changing the dynamics of the Hopfield network from chaos to convergence. In stark contrast, the basis of SSA by adding noise is stochasticity, regardless of the origin of the noise, e.g., from devices, circuits, or software. As a result, the searching process of CSA is controlled by the bifurcation of the whole dynamic system, while that of SSA is decided by random fluctuations. Consequently, CSA searches for the possible solution in a lower dimensional fractal space rather than in the entire state space, potentially leading to higher efficiency (*40*). To evaluate the difference in detail, we have performed a simulation to compare SSA and CSA, and the results reveal that CSA shows faster convergence speed and gets to lower Hopfield energy (fig. S7), hence demonstrating the advantage of CSA.

The above results in Fig. 5 show that the intrinsically nonlinear depression curve of Ta/TaO*_x_*/Pt devices when applying identical voltage pulses can naturally serve as an excellent annealing strategy, as demonstrated by its excellent performance in achieving high probability of global optimum and low iteration cycles. Therefore, it is no longer necessary to tune the pulse parameters to achieve alternative annealing processes. Simple operation and efficient annealing can be achieved at the same time with the LTD annealing, hence offering a promising route toward solution of optimization problems with transiently chaotic networks.

### Summary

In summary, we have implemented a memristor-based optimization hardware that uses transient chaos in searching for global optimum of continuous and combinatorial optimization problems. This transiently chaotic annealing is realized by gradually resetting the devices in the diagonal positions of the memristor crossbar, serving as self-feedback weights of Hopfield neurons, without introducing additional devices or circuit modules, therefore implying improved efficiency. Furthermore, the intrinsic nonlinear depression characteristics of TaO*_x_* devices can serve as an efficient and simplified annealing strategy, which can be achieved using simple pulse schemes. The potential of the transiently chaotic memristor optimizer has been experimentally demonstrated in both continuous optimization and combinatorial optimization problems, but the applicability of the approach may be extended to a large variety of optimization problems. This work could thus be of great significance for extending the capability of memristive systems from neural network accelerators to solving complex, real-world computation problems that are challenging for conventional digital computers.

## MATERIALS AND METHODS

### Mapping the algorithm onto memristor array

The algorithm is mapped onto a single memristor array, where VMM operation is accelerated by the array based on physical laws, and the diagonal devices represent the self-feedback weights. Note that in Eq. 2, the input to the self-feedback connection is different from that to the other synaptic weights by a scaling factor α and a constant parameter *I*_0_. This leads to a mismatch when the algorithm is mapped onto the array because of the fact that, in the memristor crossbar, the top electrode of the same row is physically connected, and therefore, the input to the specific row must be the same. However, if the self-feedback weights and the rest are programmed onto two separated arrays, it will cause double area and peripheral circuit consumptions. To address this issue and map the matrix parameter together with the decaying self-feedback onto a single array, we performed a mathematically equivalent transformation to the model in Eq. 2 as follows

$$y_{i}(t+1)=ky_{i}(t)+\alpha (\sum_{j=1}^{n}w_{\mathrm{ij}}X_{j}(t)+I_{i})+\alpha I_{0}\sum_{j=1}^{n}w_{\mathrm{ij}}$$ (7)

$$X_{j}(t)=x_{j}(t)-I_{0}$$ (8)

Therefore, the weight matrix including the self-feedback programmed onto the crossbar is

$$W=(\begin{array}{ccc} \frac{-z_{1}(t)}{\alpha } & \ldots & w_{1n}\\ \vdots & \ddots & \vdots \\ w_{n1} & \ldots & \frac{-z_{n}(t)}{\alpha } \end{array})$$ (9)

The input applied to each row is now replaced by *X_j_*(*t*). The above weight matrix is, in turn, programmed physically to the array according to a linear relation *G_ij_* = *aw_ij_* + *b*. Therefore, the reduction of the conductance in diagonal positions is equivalent to the weakening of self-feedback. It is also worth mentioning that although an extra summation term is needed after the mathematical transformation, the most computationally intensive part is still the VMM in the second term, which can be calculated by the memristor array in a single read operation. A network of size *n* with *n_t_* iterations needs about (2*n*^2^ − 1)*n_t_* operations (add and multiplication) from the second term, whereas the rest in Eq. 7 only requires about *n*^2^ + *n_t_* − 1. Therefore, the array can accelerate a majority of the operations.

### Memristor array fabrication

The memristor array used in this work was fabricated on SiO_2_/Si substrates. The bottom electrodes were patterned using photo lithography and subsequently formed by depositing 30-nm Pt along with 5-nm Ti via e-beam evaporation and doing a lift-off process. Afterward, a resistive switching layer of Ta_2_O_5_ (30 nm) was formed by magnetron sputtering. The Ta_2_O_5_ layer was patterned via photo lithography and lift-off. Last, the top electrode consisting of 10-nm Ta and 30-nm Pt protection layer was formed by photo lithography, magnetron sputtering, and lift-off.

### Electrical measurements

For the demonstrations based on 2 × 2 memristor array, the crossbar was directly connected to an Agilent B1500 semiconductor parameter analyzer through a probe station. Voltage pulses were generated by B1500 and applied to the rows (top electrodes) of the array, and the currents were sampled and accumulated through the columns. Other neural network operations other than VMM were programmed and implemented in Agilent Easy Expert software.

### Materials characterization

The TEM samples in this work were prepared by focused ion beam (FIB) technique using a dual-beam FIB system (FEI Helios NanoLab workstation). The microstructure of the devices was characterized by scanning TEM and EDS measurements using an FEI Tecnai F20 transmission electron microscope.

### Simulation

To compare different annealing strategies, we conducted a series of simulations. In these simulations, the classic TSP was chosen as the benchmark. We analyzed the averaged probability of converging to the global minimum and averaged iteration cycles to evaluate the efficiency of the different annealing processes. TSP is an NP-hard combinatorial optimization problem, where the salesman needs to find the shortest trip distance to traverse all the specified cities while avoiding any repetition. A *n*-city TSP can be solved by an *n* × *n* network, with neuron *x_ij_* referring to visit city *i* at the stop *j*. As a result, the final stabilized output of the neural network can represent a traveling route. Here, we use the mapping method and energy function in (*5*). In the present case, the energy function should contain two parts. On the one hand, necessary restrictions need to be added to ensure that the solution is valid, which means that each row (column) of the neuron matrix has one and only one element whose output is 1, corresponding to the fact that the salesman visits one city at a time and each city will be visited only once

$$E_{1}=\frac{W_{1}}{2}\{\sum_{i=1}^{n}{\left(\sum_{j=1}^{n}x_{\mathrm{ij}}-1\right)}^{2}+\sum_{j=1}^{n}{\left(\sum_{i=1}^{n}x_{\mathrm{ij}}-1\right)}^{2}\}$$ (10)

where *W*_1_ is a positive constant. If *W*_1_ is sufficiently large, the solution according to the energy minimum must be a valid one. On the other hand, the solution is expected to be the traveling plan with the shortest distance. Therefore, the distance information is included in the energy function as follows

$$E_{2}=\frac{W_{2}}{2}\sum_{i=1}^{n}\sum_{j=1}^{n}\sum_{k=1}^{n}(x_{\mathrm{kj}+1}+x_{\mathrm{kj}-1})x_{\mathrm{ij}}d_{\mathrm{ik}}$$ (11)

where *d_ik_* is the distance between city *i* and city *k*, and *W*_2_ is another positive constant. The total energy *E* of the network is thus

$$E=E_{1}+E_{2}$$ (12)

Our simulation is based on the above method for the 10-city TSP, where the city coordinates are randomly generated between (0,1). For all the annealing strategies, the parameters are set to be the same, that is *k* = 1, ε = 0.004, *W*_1_ = *W*_2_ = 1, and *I*_0_ = 0.65. The initial self-feedback weight *z*_0_ = 0.08 and the membrane potentials are randomly initialized in the range of ( − 1,1). For linear annealing, the decreasing self-feedback is described by *z*_lin_ = *ct* + *d*, where parameter *c* is used to control the annealing speed, and β is the cooling parameter for exponential annealing. In the LTD annealing strategy, the data can be fitted by a double exponential equation, and we vary the mapping coefficient to change the annealing speed. All simulations are performed in MATLAB environment.

## Supplementary Material

aba9901_SM.pdf

## SUPPLEMENTARY MATERIALS

Supplementary material for this article is available at http://advances.sciencemag.org/cgi/content/full/6/33/eaba9901/DC1
