# Supplementary material for: Transiently chaotic simulated annealing based on intrinsic nonlinearity of memristors for efficient solution of optimization problems
Source: Sci Adv. 2020 Aug 14;6(33):eaba9901. doi: 10.1126/sciadv.aba9901 (PMC7428342; doi:10.1126/sciadv.aba9901)
Supplement: aba9901_SM.pdf [file aba9901_SM.pdf]

Science Advances AAAS

advances.sciencemag.org/cgi/content/full/6/33/eaba9901/DC1

Supplementary Materials for

Transiently chaotic simulated annealing based on intrinsic nonlinearity of memristors for efficient solution of optimization problems

Ke Yang, Qingxi Duan, Yanghao Wang, Teng Zhang, Yuchao Yang*, Ru Huang*

*Corresponding author. Email: yuchaoyang@pku.edu.cn (Y.Y.); ruhuang@pku.edu.cn (R.H.)

Published 14 August 2020, *Sci. Adv.* **6**, eaba9901 (2020)
DOI: 10.1126/sciadv.aba9901

This PDF file includes:

Figs. S1 to S7

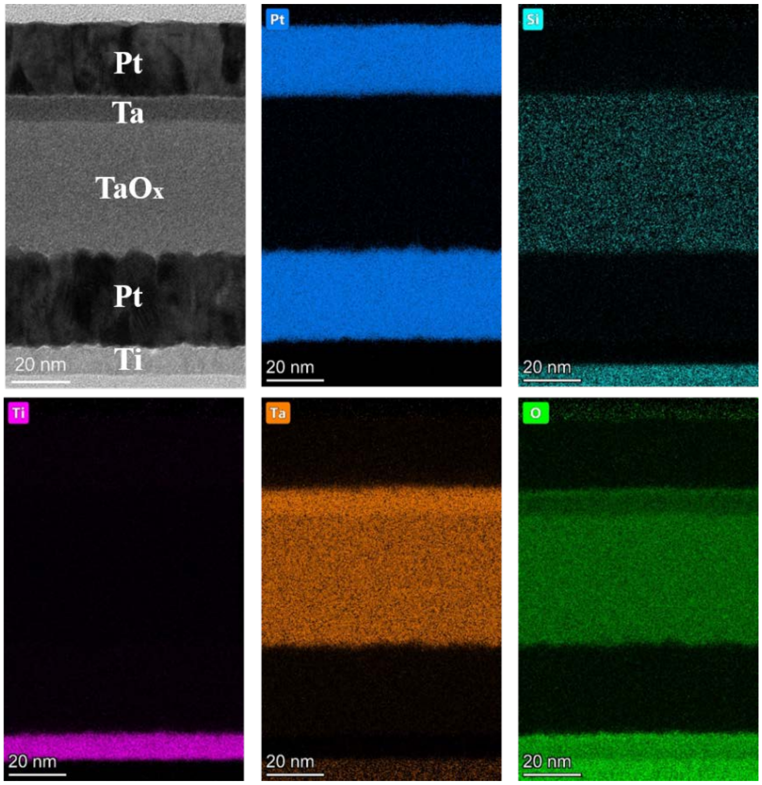

Fig. S1. Cross-sectional STEM image and compositional mapping of Ta/TaO_x/Pt devices. The STEM image and EDS mapping results clearly show the stacking structure and distributions of Pt, Si, Ti, Ta and O elements in the Ta/TaO_x/Pt device.

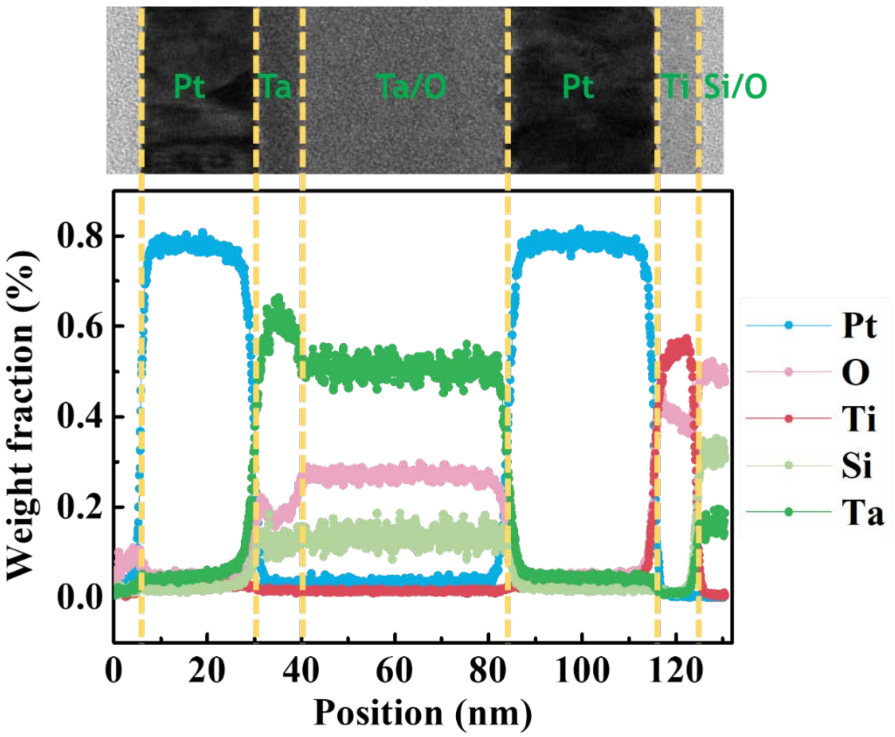

Fig. S2. Energy-dispersive X-ray spectroscopy line profile analysis of Ta/TaO$_x$/Pt device. The EDS line profile results clearly show the stacking structure and distributions of Pt, Si, Ti, Ta and O elements in the Ta/TaO$_x$/Pt device.

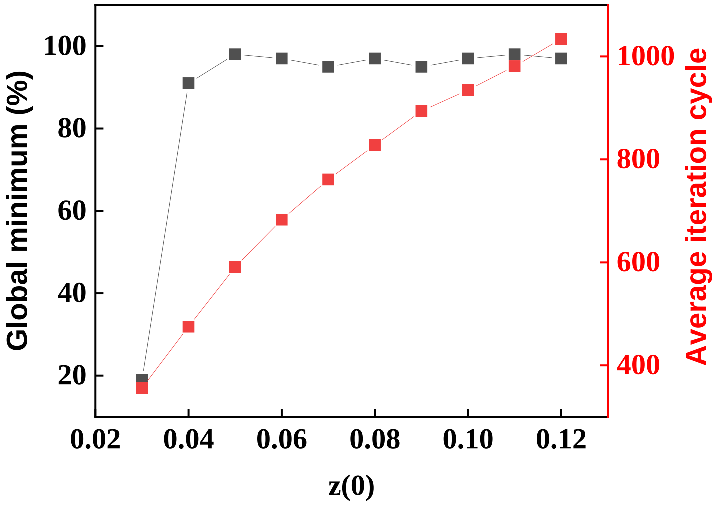

Fig. S3. Influence of different initial values of self-feedback weight on optimization. In general, the self-feedback weights need to be large enough in the beginning to ensure sufficient chaotic searching. Afterwards, the self-feedback weight is decreased according to pre-defined annealing curve (decay curve). A suitable initial value for the self-feedback weight (z_0) is thus important for the optimization. If the initial value is too small, the network will not be able to perform sufficient chaotic searching, whereas too large an initial value may prolong the convergence process to a solution. One can see that the probability of finding the global minimum is very low when $z_0 < 0.04$ and gets saturated when z_0 is 0.05–0.12. Consequently, a median value in this range, i.e. 0.08, is set to z0 in our study, which will be programmed into the conductance of diagonal devices through a linear transformation, i.e. $G_0 = a \times z_0 + b$, where a and b are decided by the dynamic range of the devices.

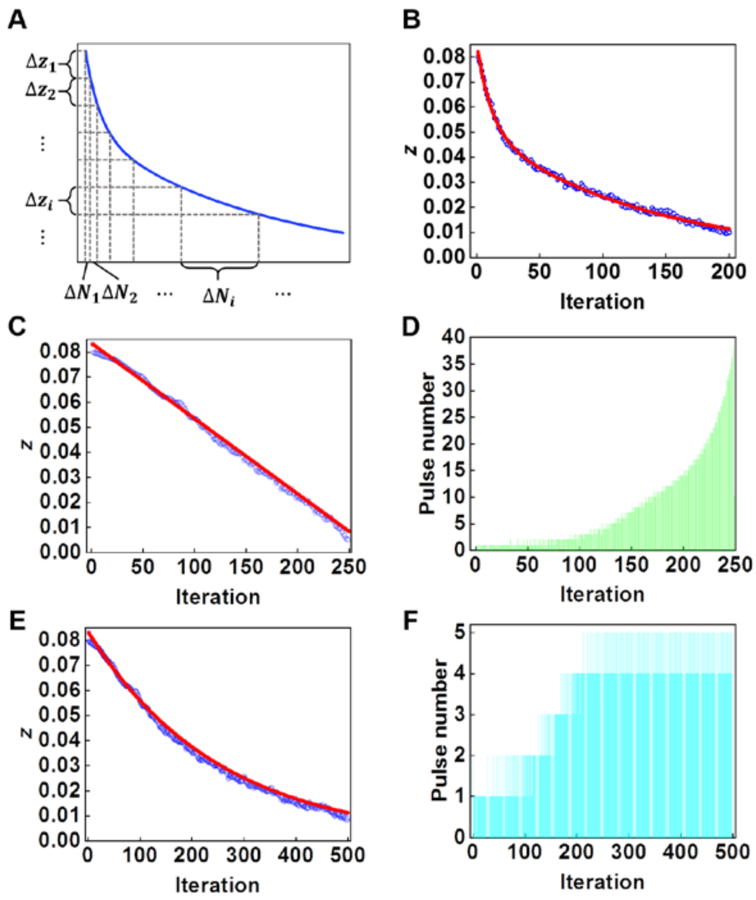

Fig. S4. Tuning the annealing process based on device dynamics. (**A**) Schematic illustration of the method. Based on known device characteristics in response to identical voltage pulses, any desired decay curved can be realized in principle. The number of voltage pulses (with fixed amplitude and width) applied at each step can be decided by looking up the device response curve, which effectively maps the target update in self-feedback weight to the number of pulses required in each step. (**B**) Long-term depression (LTD) of $Ta/TaO_x/Pt$ device when identical voltage pulses are applied, along with fitting results by $a \times e^{-bx} + c \times e^{-dx}$ (red line). (**C**) Simulation result of a linear annealing curve realized by this approach. (**D**) The pulse scheme used to implement the linear curve in (**C**). (**E**) Simulation result of an exponential annealing curve realized by this approach. (**F**) The pulse scheme used to implement the exponential curve in (**E**). However, both the linear and exponential annealing processes require modulation on the number of voltage pulses, and hence the LTD annealing is favorable in the sense of simplicity.

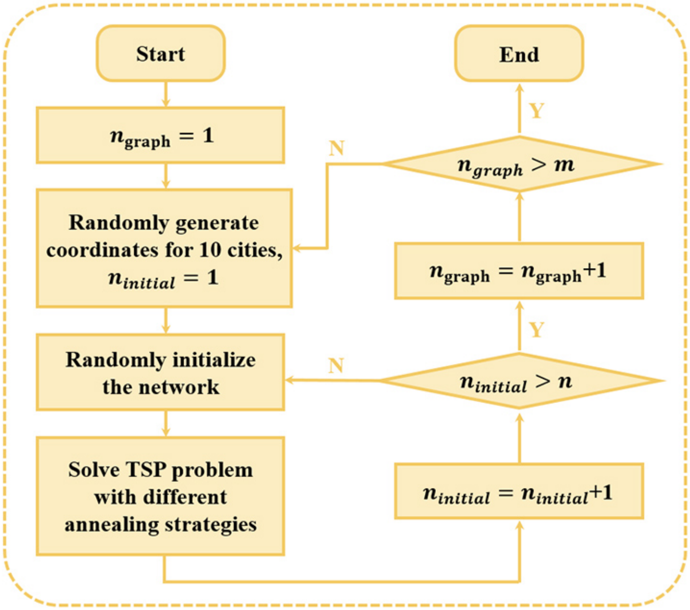

Fig. S5. Flow chart of simulation on randomly generated TSP graphs. The simulation is performed on 10 randomly generated city graphs, where the coordinates of the cities are random values between 0 and 1, following uniform distribution, and each simulation for a specific TSP problem was performed for 100 different initial conditions, namely 1000 rounds for each annealing strategy.

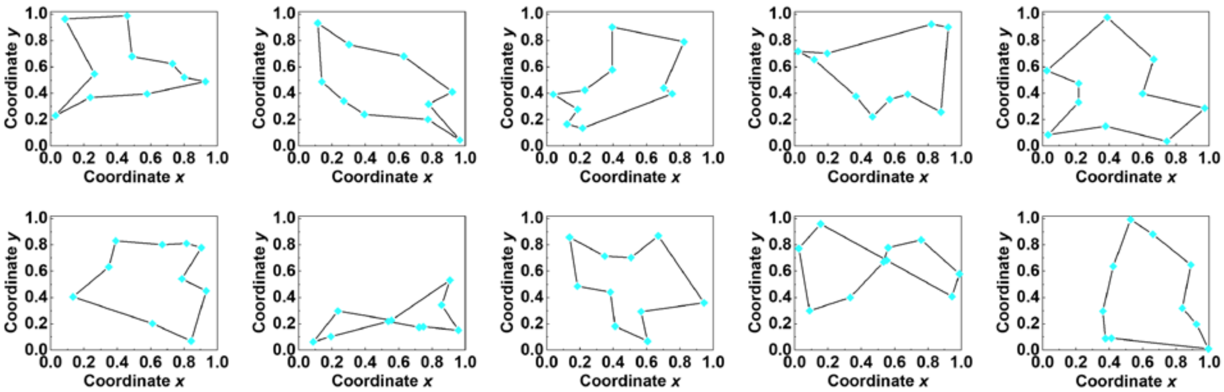

Fig. S6. Randomly generated city graphs during the simulation. The coordinates of the cities are randomly generated between (0, 1).

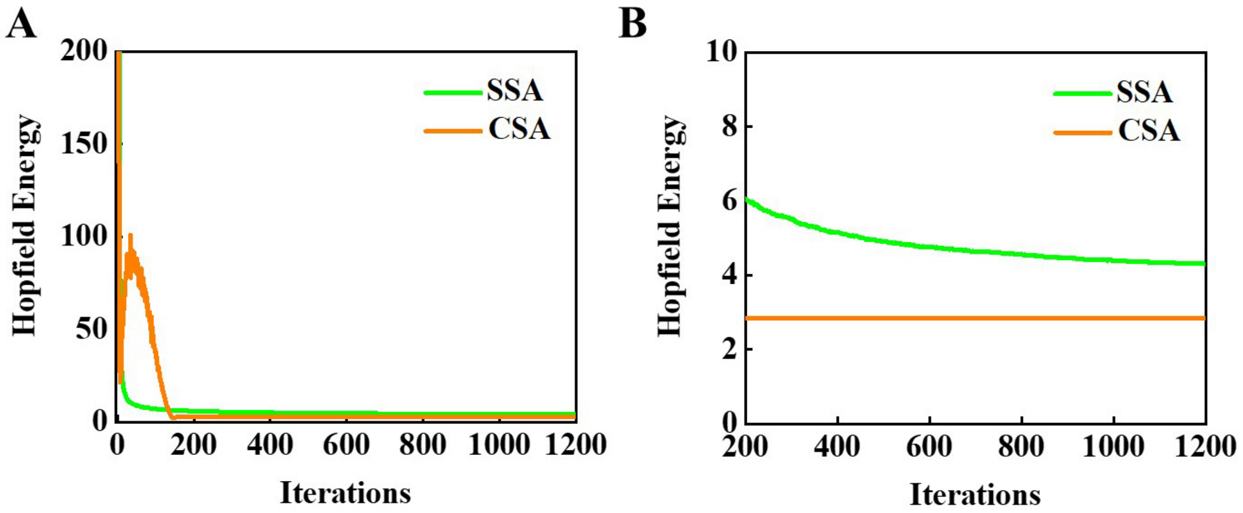

Fig. S7. Comparison between chaotic and stochastic annealing strategies. (**A**) Simulation result of averaged energy evolution when CSA (orange) and SSA (green) strategies were employed. The Hopfield energy is averaged over 100 runs with random initial conditions for 10 randomly generated TSP graphs. (**B**) A zoomed-in part of (**A**), showing that CSA has faster convergence speed and gets to lower Hopfield energy. The simulations were conducted on 10 randomly generated city graphs, with 100 randomly selected initial conditions ranging from –1 to 1 for each TSP graph. The Hopfield energy is averaged over 1000 runs. Linear annealing is employed for both CSA and SSA, and a noise item is introduced into the state of neuron in SSA before the neuron activation by Sigmoid function. The amplitude of the noise in the beginning is 0.4 and decays by 0.0005 in each time step.
